# Supplementary material for: Rapid turnover of life-cycle-related genes in the brown algae
Source: Genome Biol. 2019 Feb 14;20:35. doi: 10.1186/s13059-019-1630-6 (PMC6374913; doi:10.1186/s13059-019-1630-6)
Supplement: Supplementary file 3 — Protein alignments, corresponding Gblocks html file and CDS sequences for the lineage conserved gametophyte-biased genes. (ZIP 2109 kb) [file 13059_2019_1630_MOESM3_ESM.zip › Con_GA_alignments/Ec-12_005600.aa_ali.fasta-gb.htm]

Ec-12\_005600.aa.fasta.trans.aa\_ali.fasta


## Gblocks 0.91b Results

Processed file: **Ec-12\_005600.aa.fasta.trans.aa\_ali.fasta**  
Number of sequences: **2**  
Alignment assumed to be: **Protein**  
New number of positions: **433** (selected positions are underlined in blue)

```
                         10        20        30        40        50        60
                 =========+=========+=========+=========+=========+=========+
Ec-12_005600     MSSTAMDDLNHLETHVRLCRDGQDADAGCAETLFGNDYYQIPCTTRSRVRAIDERWEEVN
MSTRG.49951.1    ------------------------------------------------------------
                                                                             


                         70        80        90       100       110       120
                 =========+=========+=========+=========+=========+=========+
Ec-12_005600     VRATNTLDNIEEDDLDEKDPGRKRRSSNPVIDKRREAARKFSLRGTHERPPTKMLEISGA
MSTRG.49951.1    --------------------------------------------GTHESPPANISQRSHA
                                                             ################


                        130       140       150       160       170       180
                 =========+=========+=========+=========+=========+=========+
Ec-12_005600     TAWRNDIGDSGRAVTRDERVAMALAQSHENLAAAAVRFEESQERSSHLLGGLRGWLDRFT
MSTRG.49951.1    TTWLNDIGDSGRAVNRDERVAMALAQSHENLSAAAVRFEESQERSNHLLGGLRGWLDRFT
                 ############################################################


                        190       200       210       220       230       240
                 =========+=========+=========+=========+=========+=========+
Ec-12_005600     GTARPEDINPIAAMLPEAGLEDEVVALMTDCQDAALMLEHSTNRHTEQLREIFCQAGKLY
MSTRG.49951.1    GTARPEDTNPIAAMLPEPGLESEVVALMTDCQDAALMLENSTNRHTQQLREIFGQAGKLY
                 ############################################################


                        250       260       270       280       290       300
                 =========+=========+=========+=========+=========+=========+
Ec-12_005600     GSEIDKVQKKGSAASGAAREKIAGLQEHANKVEALLQSMQDERANLHTKFSGAEQRCRWL
MSTRG.49951.1    GSEMDKVQKKGSAASHAAREKINSLQEHANKVECLLQSMQDERASLHTKFSGAEQRCRWL
                 ############################################################


                        310       320       330       340       350       360
                 =========+=========+=========+=========+=========+=========+
Ec-12_005600     EERYREDKTRLQEGISNYR-------------------------EPRLAPLLDKLKAPAS
MSTRG.49951.1    EERYREDKSRLQEGLSSYRHQVVQLQHVVAGYEEKARKQEQIEREPRLAPLLDKLQPPAP
                 ###################                         ################


                        370       380       390       400       410       420
                 =========+=========+=========+=========+=========+=========+
Ec-12_005600     ATDAGQLRQLQAETARLSTENAALTEANKKLYRDLVVLRLADQGGKRSGRRGSTFAPSDL
MSTRG.49951.1    AANAEQLRTLQAETTRLASENAALTQANKKLYRDLIVSRLADQGGKRAAGRRST--RADM
                 ######################################################    ##


                        430       440       450       460       470       480
                 =========+=========+=========+=========+=========+=========+
Ec-12_005600     DTLSETSTASVRDD----RSNNAAAEQTLRNAREQSAQAEKALLESKRENELLTTRLQSL
MSTRG.49951.1    ESLSEASTTSVADDGGGTAAPDTAAKEMLRAAREKSAQAEKALLDSKRENEALVSKLQNL
                 ##############         #####################################


                        490       500       510       520       530       540
                 =========+=========+=========+=========+=========+=========+
Ec-12_005600     SAELEKEKNKKATTVVNSPAPDAVEEGLRGAIRDLRRQSSGQLSEEVEARRKAVGELEAA
MSTRG.49951.1    NVELEKEKKK--PAVINRPAPDAVEEGLRGAIRDLRRQSSGHLSDEIEARRKAVGELEAA
                 ##########    ##############################################


                        550       560       570       580       590       600
                 =========+=========+=========+=========+=========+=========+
Ec-12_005600     LEQARKKEVAAAEANAELSNLRLELAKTQEQVQALHESAAT-AKALADAS-ASLPPEPLP
MSTRG.49951.1    LQQAQQKEIAAAEANAELLKLRSELIKTQEEVQALNQSAVEAAKVRSDASRDSPPV----
                 #######################################                     


                        610       620       630       640       650       660
                 =========+=========+=========+=========+=========+=========+
Ec-12_005600     LSEATEAPAAETTTPSRDTSEEINILQPVDYDGLVDRALSQEKVGLGELRRKAKDAGVTQ
MSTRG.49951.1    ------------------------------------------------------------
                                                                             


                        670       680       690       700       710       720
                 =========+=========+=========+=========+=========+=========+
Ec-12_005600     RHCDKADKMFSDVVDCCGNGLSSVESILEGTKTIVDEDASSIQEALAAALERAIASSSVL
MSTRG.49951.1    ------------------------------------------------------------
                                                                             


                        730       740       750       760       770       780
                 =========+=========+=========+=========+=========+=========+
Ec-12_005600     LKEALVGRIRACEAEKKVSLEQEAAEEERAKAVALSQENMVLVDELQQHLVQVRTLRKEV
MSTRG.49951.1    ----------------------------------------------EPHLIPV-------
                                                                             


                        790       800       810       820       830       840
                 =========+=========+=========+=========+=========+=========+
Ec-12_005600     GALRQENQHARLQQQHQEEMAEAAGGMHAQDAEANPPPHEEVIDIAAPPSPHAAAEEEPP
MSTRG.49951.1    ------------------------------------------------------ASETPP
                                                                             


                        850       860       870       880       890       900
                 =========+=========+=========+=========+=========+=========+
Ec-12_005600     PTVVADDTGTWRRRYEHLAGLLAVQTKQNSAMASRLATSGRTGPSSSRRSSPRRRIVPPA
MSTRG.49951.1    P-----------------------------------------------------------
                                                                             


                        910       920       930       940       950       960
                 =========+=========+=========+=========+=========+=========+
Ec-12_005600     ETPLSLGTPTASPEERLPHSTGPSTAVSKNGGAKPRSSPKRANRSSNTSPRPPSPAIAEV
MSTRG.49951.1    ------------------------------------------------------------
                                                                             


                        970       980       990      1000      1010      1020
                 =========+=========+=========+=========+=========+=========+
Ec-12_005600     SGQEVGTGSRATLVSTGVSASNSPPPLVDTATTVGVSDVRVGAPRKASVDSGVQVGGGPT
MSTRG.49951.1    -------------------ATAAPS-----------------------------------
                                                                             


                       1030      1040      1050      1060      1070      1080
                 =========+=========+=========+=========+=========+=========+
Ec-12_005600     QPVVETSDGGTQADYDGVATTNDADVPVSPVVALAQPSDSSNAESNNGNPAVEQPEPSKA
MSTRG.49951.1    -----------------------------PV-----------------------------
                                                                             


                       1090      1100      1110      1120      1130      1140
                 =========+=========+=========+=========+=========+=========+
Ec-12_005600     PFVEAAETQSTAAAEDKSKGITAEEESNGTLQAQEVAEGSDNRADQDGSASTTAIMEATD
MSTRG.49951.1    ------------------------------------------------------------
                                                                             


                       1150      1160      1170      1180      1190      1200
                 =========+=========+=========+=========+=========+=========+
Ec-12_005600     GMVPAKRPETVEDPRGDGALEPEREEQVAGSSIVETRETRDTSATTNGEGGKENGGKEEG
MSTRG.49951.1    ------------------------------------------------------------
                                                                             


                       1210      1220      1230      1240      1250      1260
                 =========+=========+=========+=========+=========+=========+
Ec-12_005600     DEVMEHGERMENRVQDDDDTDHRSTDSRGSRGCDTSTASPTDAGPDGRGRDRYESIDGEA
MSTRG.49951.1    ------------------------------------------------------------
                                                                             


                       1270      1280      1290      1300      1310      1320
                 =========+=========+=========+=========+=========+=========+
Ec-12_005600     LLGKLLDEETPSAAFEDDHQKLLHKLMRVRDDEYERDKVSAQSSLSSSSSSTECLECPVL
MSTRG.49951.1    ------------------------------------------------------------
                                                                             


                       1330      1340
                 =========+=========+===
Ec-12_005600     RESLAERSAEVENAREQARKLTC
MSTRG.49951.1    ----------------------T
```

```
Parameters used
Minimum Number Of Sequences For A Conserved Position: 2
Minimum Number Of Sequences For A Flanking Position: 2
Maximum Number Of Contiguous Nonconserved Positions: 8
Minimum Length Of A Block: 10
Allowed Gap Positions: None
Use Similarity Matrices: Yes
```

```
Flank positions of the 5 selected block(s)
Flanks: [105  319]  [345  414]  [419  434]  [444  490]  [495  579]  

New number of positions in Ec-12_005600.aa.fasta.trans.aa_ali.fasta-gb:  433  (32% of the original 1343 positions)
```
